# Standardising Home Range Studies for Improved Management of the Critically Endangered Black Rhinoceros

**DOI:** 10.1371/journal.pone.0150571

**Published:** 2016-03-30

**Authors:** Roan D. Plotz, W. James Grecian, Graham I.H. Kerley, Wayne L. Linklater

**Affiliations:** 1 Centre for Biodiversity and Restoration Ecology, School of Biological Sciences, Victoria University of Wellington, Wellington, New Zealand; 2 Institute of Biodiversity, Animal Health and Comparative Medicine, College of Medical, Veterinary and Life Sciences, University of Glasgow, Glasgow, Scotland, United Kingdom; 3 Centre for African Conservation Ecology, Department of Zoology, Nelson Mandela Metropolitan University, Port Elizabeth, South Africa; University of Illinois at Urbana-Champaign, UNITED STATES

## Abstract

Comparisons of recent estimations of home range sizes for the critically endangered black rhinoceros in Hluhluwe-iMfolozi Park (HiP), South Africa, with historical estimates led reports of a substantial (54%) increase, attributed to over-stocking and habitat deterioration that has far-reaching implications for rhino conservation. Other reports, however, suggest the increase is more likely an artefact caused by applying various home range estimators to non-standardised datasets. We collected 1939 locations of 25 black rhino over six years (2004–2009) to estimate annual home ranges and evaluate the hypothesis that they have increased in size. A minimum of 30 and 25 locations were required for accurate 95% MCP estimation of home range of adult rhinos, during the dry and wet seasons respectively. Forty and 55 locations were required for adult female and male annual MCP home ranges, respectively, and 30 locations were necessary for estimating 90% bivariate kernel home ranges accurately. Average annual 95% bivariate kernel home ranges were 20.4 ± 1.2 km^2^, 53 ±1.9% larger than 95% MCP ranges (9.8 km^2^ ± 0.9). When home range techniques used during the late-1960s in HiP were applied to our dataset, estimates were similar, indicating that ranges have not changed substantially in 50 years. Inaccurate, non-standardised, home range estimates and their comparison have the potential to mislead black rhino population management. We recommend that more care be taken to collect adequate numbers of rhino locations within standardized time periods (i.e., season or year) and that the comparison of home ranges estimated using dissimilar procedures be avoided. Home range studies of black rhino have been data deficient and procedurally inconsistent. Standardisation of methods is required.

## Introduction

Accurate home range estimates are important because they provide insight into the ecological needs of an organism and the spatial structure of populations [1]. The home range size of an animal will vary amongst sites and through time as a consequence of differences and changes in population demography (e.g., density, sex ratio) and resources (e.g., water, food, shelter). Home range data, therefore, is relied on for conservation planning and monitoring efforts towards species recovery. However, the reliability of home range size estimates and their usefulness as a conservation tool has been eroded by the tendency for studies to ignore minimal data requirements and use dissimilar techniques that prevent meaningful inter-study comparisons [2, 3]. There have been, therefore, recent calls for studies to address sources of error in home range methodology and interpretation [1, 4].

Home range size has been an important part of management decision-making for the critically endangered black rhinoceros [5], but studies (n = 24) have been plagued by data deficiencies, undescribed methodological detail and inconsistent estimations of home ranges (S1 Table, [3, 6]). The historical home range estimates for black rhino in Hluhluwe-iMfolozi Park (HiP), South Africa (Table 1), illustrates these procedural inconsistencies and the potential for home range studies to mislead conservation management [3]. In HiP, an alleged 54% increase in black rhino home range estimates in different studies from 1991 to 2001 ([7]cf. [8]) was used as evidence for deteriorating habitat quality and over-population [8]; but the home ranges in each study were calculated using different methods, with differing number of rhino locations collected over varying periods of time. Standardisation guidelines for black rhino home range studies are required.

**Table 1:** Detailed comparison of methodology of home range studies of black rhinoceros *Diceros bicornis minor* in Hluhluwe-iMfolozi Park, South Africa.

	Hitchins 1969 [45]	Hitchins 1971 [46]	Adcock 1996 [53] and Emslie 1999 [7]	Reid et al. 2007 [8]	This study
**Data collection**	Ground search, fortuitous observations	Radio-telemetry	Fortuitous observations	Fortuitous observations	Radio-telemetry, random stratified approach
**Data analysis**	Visual approximation using all points	Visual approximation using all points	1km^2^ grid occupancy data	95% adaptive KUDs*	95% MCP*, 50%, 90%, 95% bivariate KUDs* and Hitchin’s [45–46] visual approximation technique using all points
**Observation period**	< 1 year (1962–63)	From 3–13 months (Nov. 1969-Dec. 1971)	c. 4 years** (c. 1991–94)	c. 11 years** (c. 1991-Feb. 2002)	Annual (consecutive 12-months) and seasonal (wet: Oct—Mar, dry: Apr—Sep) between Jan. 2004-Dec. 2009
**Locations per rhino**	Not reported	47–503	~6–20	≥ 10	30–80 annually and 25–47 per wet and dry season
Sampling frequency***	Not reported	Twice daily	Not reported	< 1 per year	Average 6.3 ± SE 0.4 days (range: 1–49)
**Focal population**	4f, 2m	4f, 10m	Not reported	125	18f, 7m
**Park management sections**	Nqumeni (Hluhluwe)	Nqumeni (Hluhluwe)	Manzibomvu (Hluhluwe)	All 5 sections	Mbhuzane, Masinda (iMfolozi) and Nqumeni (Hluhluwe)

* MCP is an acronym for minimum convex polygons and KUDs an acronym for kernel utilisation distributions.

**used an 11-year data set (c. 1991–2002) that incorporated Adock’s [53] same four-year data set (1991–1994) i.e., lack of independence between data sets. Also, Reid et al. [8] did not just present home ranges from combined data over the 11-year period, but also represent seasonal ranges from data that combined locations from summer/ winter in one year with summer/ winter locations from several other years between 1991–2002 (Linklater et al. [3]).

***Average duration between locations.

The number of locations, observation period, and procedures used to construct home ranges will modify home range location and size estimates (e.g., [4, 9–11]). Standardising and comparing black rhino home range size estimates between studies will depend on understanding how the different procedures for defining home ranges influence outcomes. The time period over which those procedures are applied will also change the results. Ecologically meaningful home range comparisons depend on understanding what periods (seasons) impose changes in animal range-use pattern and sampling similarly across those periods. Reliable home range size estimates also necessitate analyses for the minimum number of animal locations required, but such calculations are seldom reported ([6], S1 Table).

In this study, we present home range data for the largest cohort (n = 25) of black rhino yet fitted with VHF radio-transmitters and monitored intensively over an extended period (2004–2009). We estimated the minimum number of locations required for accurate black rhino home range estimates over annual and seasonal time periods and constructed a 95% minimum convex polygon (MCP), and 90% and 95% bivariate kernel utilisation distributions (hereafter KUDs), home ranges for comparison with contemporary estimates in the historical sequence (Table 1). We use these data to re-evaluate the hypothesis [8] that black rhino home ranges in HiP have increased substantially in recent years. Our study aimed to motivate improvements in the accuracy of black rhino home range estimations and recommend procedural standardisations towards more meaningful comparisons across studies, especially where conservation management decisions depend on home range data.

## Methods

### Study area

Hluhluwe-iMfolozi Park (HiP) (S28° 0´ to 28° 25´, E31° 42´ to 32° 0´) is a 960 km^2^ fenced reserve located in Zululand, KwaZulu-Natal, South Africa. Mean annual rainfall and altitude decrease from Hluhluwe in the North (990 mm and 450 m asl), to iMfolozi in the South (635 mm and 60 m asl), with April to September being the dry season [12]. The temperature ranges between an average 13°C in winter and 33°C in summer [12]. During this six-year study (2004–2009) average summer (October to March) rainfall was 378.0 mm and winter rainfall 201.9 mm (HiP’s Masinda weather station; G. Clinning, Ezemvelo KwaZulu-Natal Wildlife, unpublished data). HiP has had a quasi-periodic wet-dry rainfall oscillation lasting approximately nine years [13]. During this study, the park was in a period of below average rainfall initiated in c. 2001 (see [14]). The sub-tropical vegetation varies from grasslands to *Acacia* sp. woodlands and denser thickets dominated by broadleaf species like *Euclea* and *Maytenus* [15].

HiP holds approximately 218 south-central black rhino (*D*. *b*. *minor*) [16]–the larger relict population of only two in Africa [17–18]. HiP undertakes annual black rhino harvesting (*c*. 5 to 8% of the population) as it serves as a source population for reintroduction and restocking [16, 18–21].

### Transmitter Installation Procedures

Whereas GPS telemetry has been effectively applied to other animals (e.g., satellite collars on savanna elephants, *Loxodonta africana* [22]), in black rhino radio-telemetry using horn implant transmitters is preferred because these transmitters are not subject to the detachment, overheating and breakage of the unit or its attachment, that have prevented GPS telemetry being used for black rhino [23]. From January 2004 to October 2008 black rhino were intermittently captured by a wildlife veterinarian darting from a helicopter, as part of a study of black rhino population dynamics (see [15, 24–25]). Horn implant VHF radio-transmitters were inserted into 25 black rhino (18 females, 7 males) similar to the techniques described elsewhere [26–27]. Radio-transmitters had an approximate maximum lifespan, weight, diameter and height of 1.3 years, 32 grams, 32 mm and 25 mm respectively (i.e., fits into a 34 mm hole in horn). Radio-transmitters were either Telonics (U.S.A.) or Sirtrack Pty Ltd. (NZ) models, and VHF radio-telemetry signals were recorded (frequency 148 to 174 MHz) via a TR-4 receiver (Telonics, Inc., Mesa, AZ, U.S.A.). All capture and study procedures were approved by Ezemvelo KwaZulu-Natal Wildlife (EKZNW) research department (Hill Top; permit no: ZC/101/01), Victoria University of Wellington Animal Ethics Committee (2007R2) and Zoological Society of San Diego (IACUC number 169).

### Location data

We located rhinos fitted with radio-transmitters from 2004 to 2009. All study rhino were regularly located (average once every 6.3 ± SE 0.4 days, range: 1–49) at irregular intervals between dawn and dusk. We relocated all radio-transmittered study rhino in random sequence without replacement before restarting the process, thus rhino accessibility did not bias relocation data. Data collection for each rhino consisted of alternating a rhino’s location sequence between direct sightings (an observation) and triangulation estimates. For example, if a rhino’s location was obtained by triangulation, the next location obtained for that rhino, when re-selected, would be a direct sighting and vice versa. To reduce temporal auto-correlation of data, we maintained a minimum period of ≥ 1-day between locations of all study rhino.

The first step leading to a direct sighting of individual rhino was obtained by determining the direction of their unique radio signals from high elevation. The rhino and its location were verified by tracking it on foot and trying to remain downwind from it until the rhino was sighted. Triangulation estimates were obtained by an observer taking bearings (i.e., measuring direction of rhino’s unique radio-transmitter signal) from two or more elevated positions ranging from 0.1 to 3.8 km from rhino. A hand-held compass was used to estimate the direction of the rhino’s radio-signal to an accuracy of 1°. Positions of direct sighting and triangulation bearing points were determined via hand-held GPS units (Garmin e-trex model). All triangulation location bearings were converted into GPS locations using Locate III software [28]. Triangulation bearings less than 60° and greater than 180° apart were excluded from estimates of rhino locations because they result in lines that intersect gradually and are known to introduce substantial location estimate error [29].

Locations over 12-months were obtained for 17 different rhino to allow annual home range estimates (average locations per rhino = 50.8 ± SE 3.4 locations; range: 30–80). For seasonal home range estimates, we gathered individual rhino locations from 25 rhino during wet (start of October to end of March; average locations per rhino = 27.6 ± SE 1.5 locations; range: 30–38) and dry (start of April to end of September; 31.3 ± SE 1.6 locations; range: 25–47) seasons over the 6-year field study.

### Home range construction and comparison

**Fig 1:**
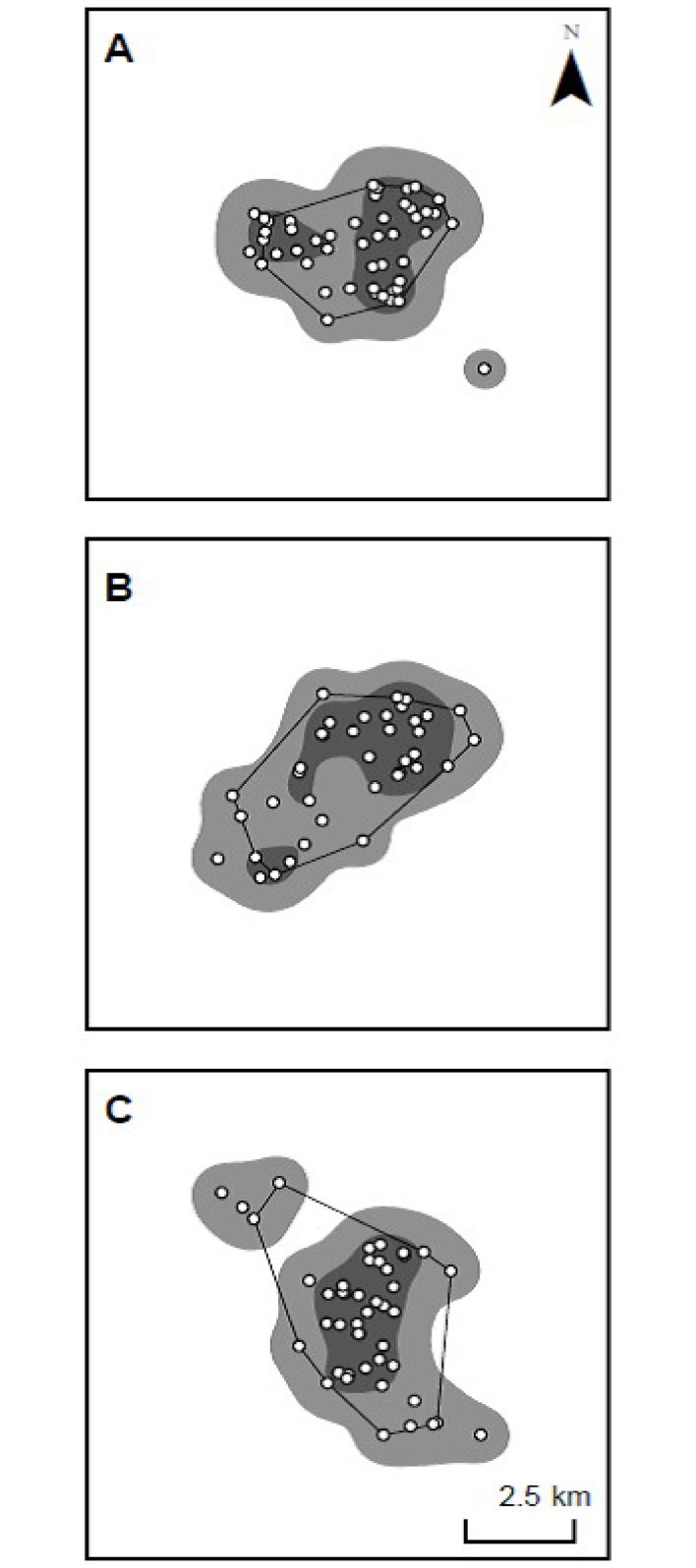
Illustrated comparison of the two analysis techniques used for producing annual black rhino home-range estimates in Hluhluwe-iMfolozi Park, South Africa. Comparisons are illustrated for the ranges of three rhinos designated A, B and C. Actual positions from radio-telemetry relocation are illustrated by the white filled circles. Contours of bivariate kernel utilisation distributions are illustrated by the 50% (dark grey) and 95% (mid-grey), using a smoothing parameter (h) of 500 m and cell size of 100 m in an African Albers Equal Area projection. 95% Minimum Convex Polygon (MCP) range estimates are illustrated by the black line polygon. Note that Rhino A and B were classified as adults (≥ 8 years), Rhino C was a sub-adult (< 8 years) and 95% kernels produced consistently larger estimates than 95% MCPs.

The home range of an individual animal is typically constructed from a set of location points that have been collected over a period of time, identifying the position in space of the individual over that time period. MCPs and KUDs are two of the most widely used metrics for wildlife home range analysis, including for black rhino (Table 1, S1 Table). MCPs completely enclose all location data points by connecting the outer locations in such a way as to create a convex polygon (Fig 1). Because MCPs are sensitive to the most extreme locations in space [30], 95% MCPs improve standardisation in animal home range calculations [31–35] by removing the 5% of extreme locations that are assumed more likely to be associated with an animal’s dispersive or investigatory movements, rather than movements for routine resources that have typically defined animal home ranges [31–35]. In contrast, KUDs are a probabilistic method first introduced to the ecological literature by Worton [36–37]. KUDs calculate home range boundaries based on the distribution of locations and takes the form of a two dimensional probability density function that represents the probability of finding an animal in a defined area within its home range ([32], Fig 1). Because KUDs are sensitive to location density, KUDs can inflate range estimates beyond park boundaries where locations occur near fence lines. Adjustments where required are needed to remove inaccessible land areas from KUD home range size estimates.

Locations estimated by triangulations and actual locations were transferred to a Geographic Information System (GIS: ArcView 10.0 and its Home Range extension, [38–39]), and plotted onto a map of HiP to see if the home range analysis techniques used (i.e., MCPs and KUDs) extended any rhino’s range size estimate beyond the park’s borders. Our analysis showed that none of our rhino’s home range size estimates exceeded the park’s fenced boundary and so no adjustments were necessary.

Average home range estimates over a 12-month (annual) period were based on 95% MCP and 90% KUD estimates. We also calculated 50% and 95% KUDs to determine the intensity of habitat use (i.e., ratio of core to total home range) and allow comparisons with other studies. Börger et al. [40] found that 50 to 90% KUDs are significantly more accurate estimators of animal home range size than 95% KUDs. Thus, 90% KUDs were also calculated to encourage future studies to strive for improved accuracy. For the smaller seasonal (6-month) datasets we constructed home ranges using only the MCP technique because they are typically more robust where location number is small (i.e., less affected by location density than KUDs, [1, 2, 40–41]). MCPs have also traditionally been the most common metric used in home range studies—facilitating comparisons with most other black rhino and animal home range studies [2, 6, 34–35, 42].

To test how many locations were needed to accurately estimate black rhino seasonal and annual home ranges we calculated 95% MCPs and 90% KUDs on incremental increases (i.e., intervals of 5) in chronologically ordered location data to determine at what location interval male and female range sizes were within 10% of their total estimated home range size (i.e., approaching the asymptote) (e.g., [2, 43–44]). Defining exactly where asymptotes begin and the number of locations required is subjective and so within 90% (i.e., within 10%) of the total home range was used as a defining threshold to determine number of locations needed (e.g., [44]. Only those individuals with a sufficient number of locations above this threshold were included for further analysis of home range size.

To allow comparison with other historical estimates of black rhino home ranges in HiP [8, 45–47], we applied Hitchin’s [45–46] technique (a subjective smoothed line drawn around the outer-most locations), 95% MCP’s, and 50% and 95% KUD contours calculated using a smoothing parameter (h) of 500 m and a cell size of 100 m to our data (Fig 1).

All MCP and KUD estimation was carried out in R-project for statistical computing [48] using the packages ‘adehabitatHR’ [49] and ‘maptools’ [50], in an African Albers-Equal Area projection calculated using ArcGIS 10 [38].

Lastly, the ratio of 50% (core) to 95% KUD home ranges was calculated to evaluate the intensity of home range use. Home ranges used more evenly should have higher ratio scores, while lower ratio scores are associated with the importance of smaller ‘patches’ of habitat within the home range [6, 34].

## Results

### Home range construction

A total of 1939 locations were obtained. Forty-seven percent (n = 906) were direct sightings and 53% (n = 1033) were triangulations (cf. [51]). Five triangulated locations were excluded because they estimated the individual to be outside of the reserve’s impenetrable fence.

**Fig 2:**
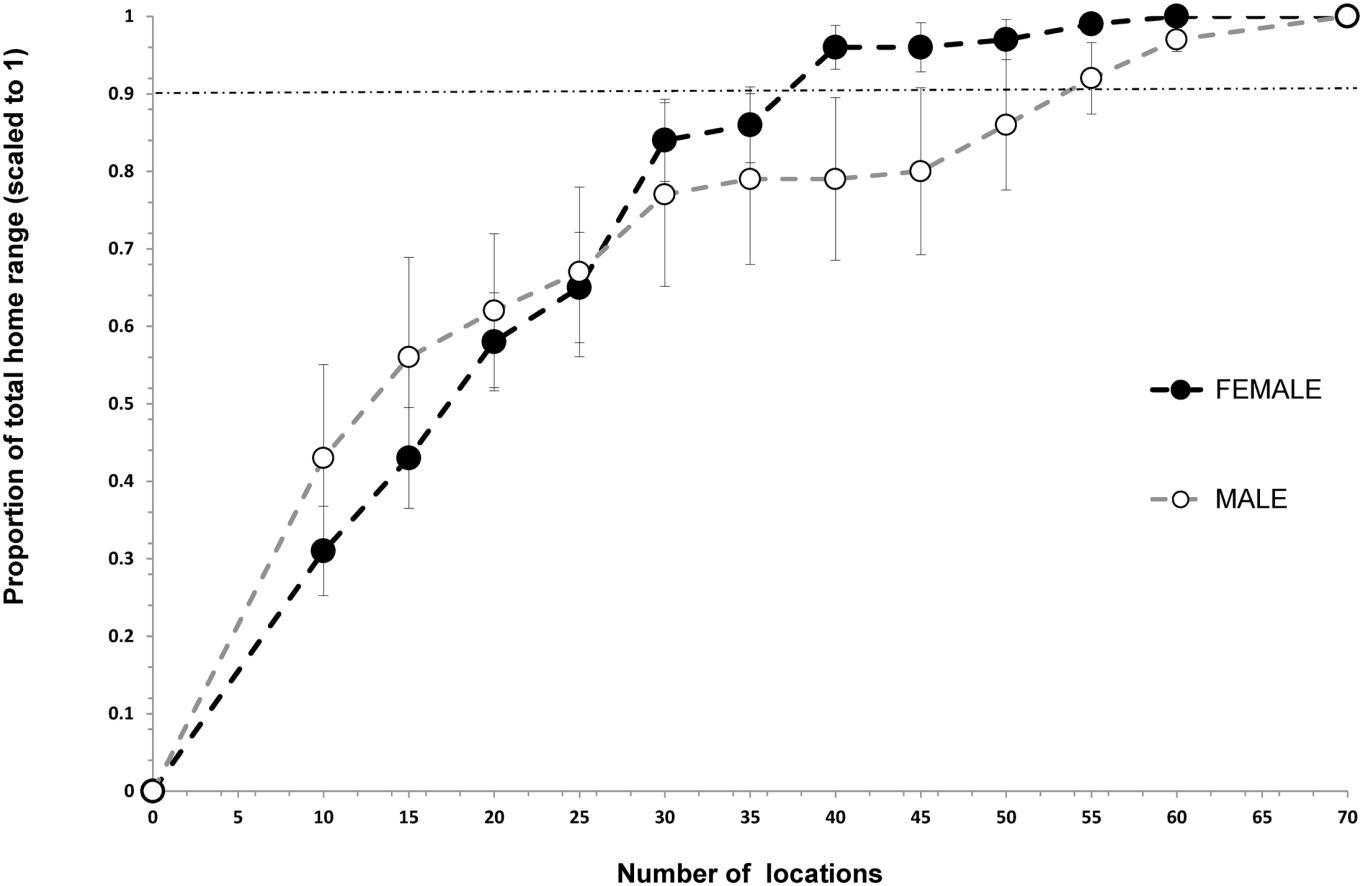
Incremental 95% MCP accumulation curve showing the number of locations required to more accurately estimate the annual (any consecutive 12-months) home ranges for black rhino in Hluhluwe-iMfolozi Park (HiP), South Africa. Note that the horizontal dashed line represents the within 10% level of the total home range recommended for increased accuracy.

**Fig 3:**
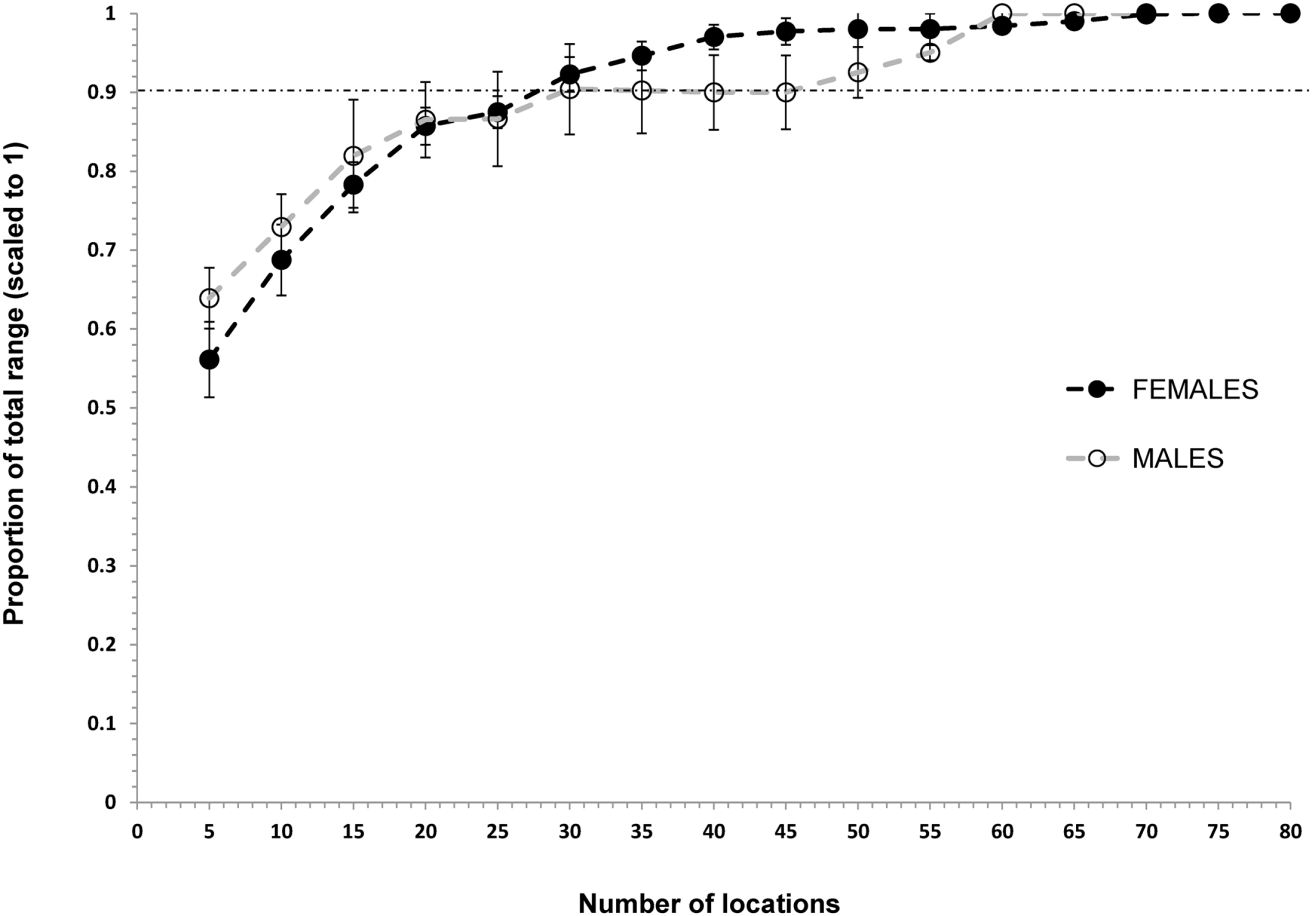
Incremental 90% KUD accumulation curve showing the number of locations needed to accurately estimate the annual (any consecutive 12-months) home ranges for black rhino in HiP. Note that the horizontal dashed line represents the within 10% level of the total home range recommended for increased accuracy.

**Fig 4:**
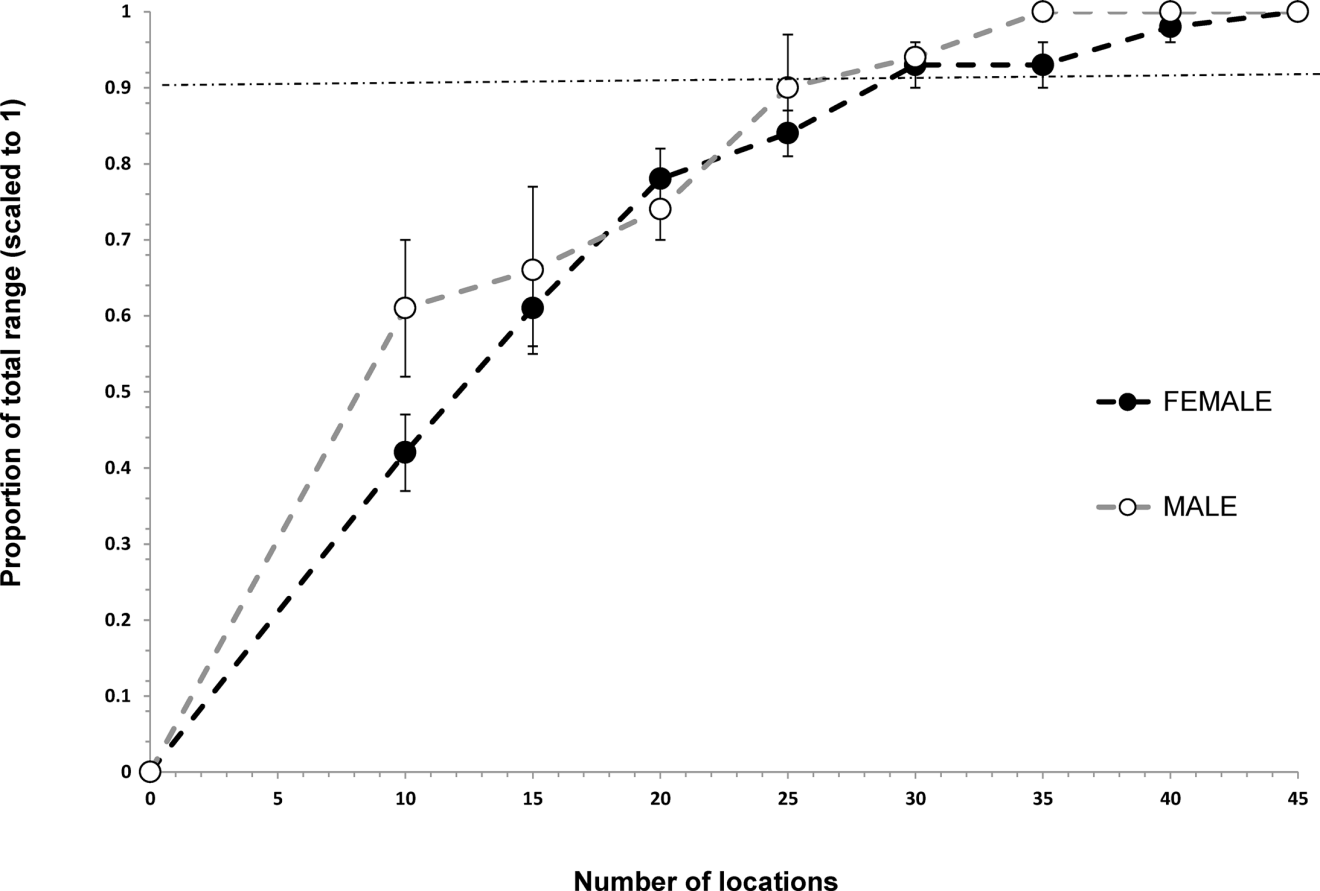
Incremental 95% MCP accumulation curve showing the number of locations required to more accurately estimate the dry season (Apr-Sep) home ranges for black rhino in Hluhluwe-iMfolozi Park (HiP), South Africa. Note that the horizontal dashed line represents the within 10% level of the total home range recommended for increased accuracy.

**Fig 5:**
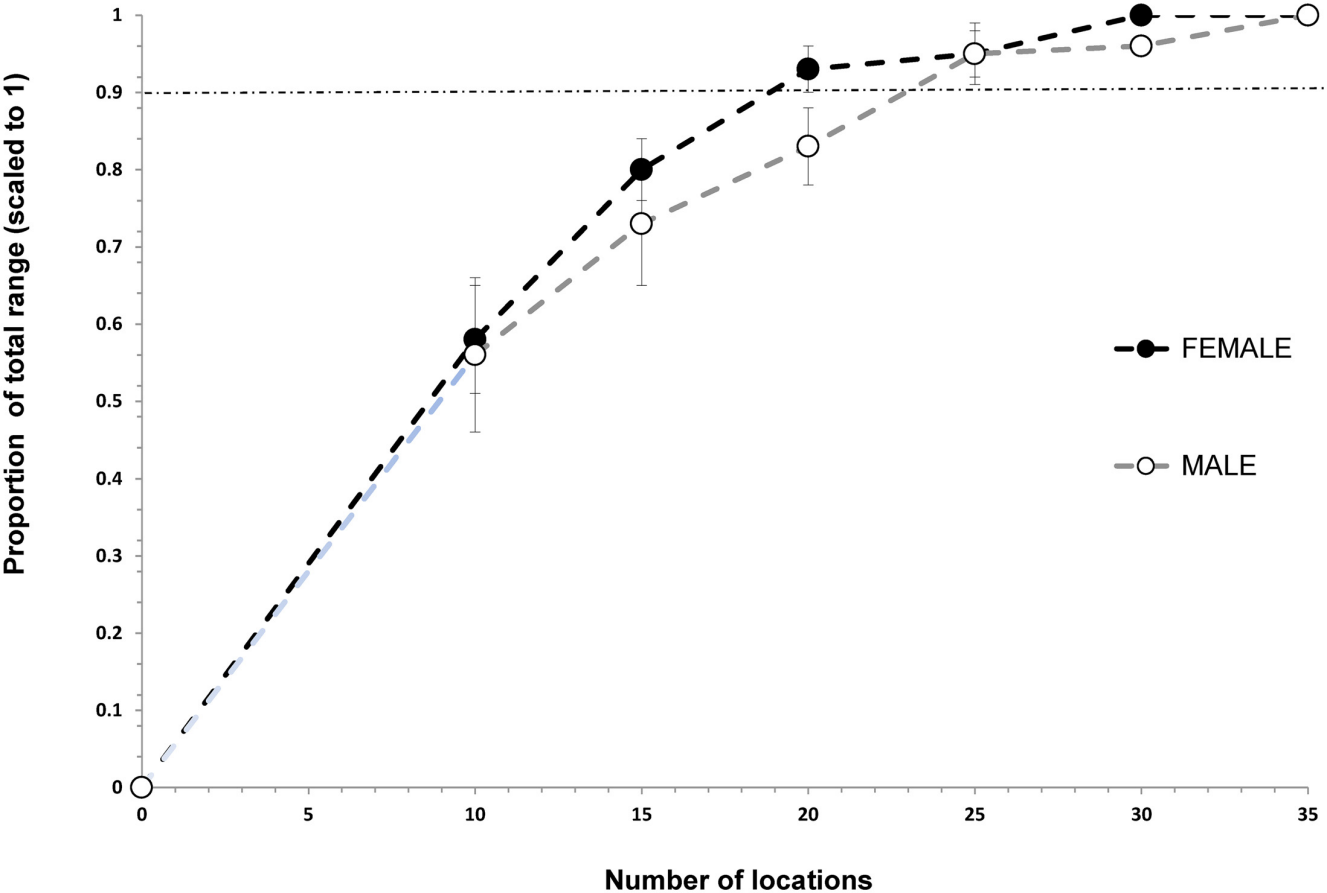
Incremental 95% MCP accumulation curve showing the number of locations required to more accurately estimate the wet season (Oct-Mar) home ranges for black rhino in Hluhluwe-iMfolozi Park (HiP), South Africa. Note that the horizontal dashed line represents the within 10% level of the total home range recommended for increased accuracy.

Asymptotic location plots showed that location requirements for annual home ranges were different between the sexes. Adult males required 55 and 50 locations to build 95% MCP and 90% KUD annual home ranges, respectively, but females just 40 and 30 locations for an accurate estimate (Figs 2 and 3). 95% MCP home range size for black rhino required a minimum of 30 and 25 locations in the dry and wet seasons respectively (Figs 4 and 5).

These values were exceeded for six males with 55 to 71 locations, and 11 females with 40 to 79 locations. Three males with 30 to 35 locations and 19 females with 30 to 46 locations exceeded the approximately 30 locations that were needed to estimate home range size during the 6-month dry season. Finally, six males with 30 to 38 locations and six females with 25 to 30 locations exceeded the minimum 20 and 25 locations needed, respectively, by the sexes, to accurately estimate wet season range size.

### Home range size

Mean annual 95% MCP home range size for females was 10.4 ± SE 1.4 km^2^ (n = 11); for males it was 8.7 ± SE 0.9 km^2^ (n = 6). Implementing and replicating the technique in references [45–46] for drawing black rhino home ranges to our data yielded similar range sizes to MCPs: females 9.9 ± SE 1.0 km^2^ (n = 11) and 9.4 ± SE 0.7 km^2^ for males (n = 6).

**Fig 6:**
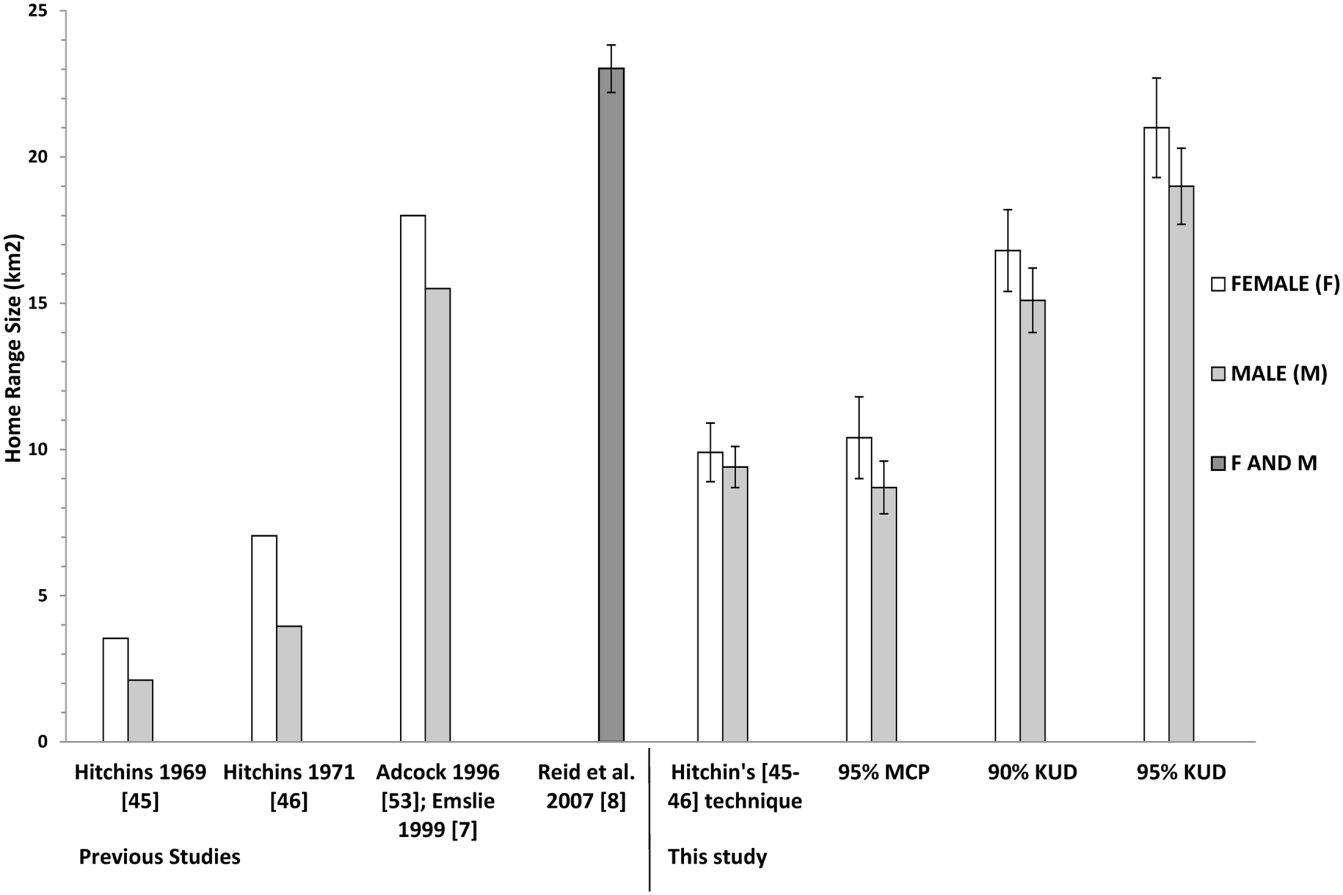
Historical sequence of home range estimates for black rhino *Diceros bicornis minor* in Hluhluwe-iMfolozi Park, South Africa. Included for comparison are the four home range estimates from this study where different analysis techniques were used (i.e., Hitchin’s [45–46] visual approximation technique, 95% MCP’s, 90% and 95% kernels). Standard error is included for home range estimates where possible. Note that the unshaded (white) bars represents average range sizes for female rhino, whereas the shaded (light grey) bar represents male averages. The darker shaded bar (dark grey) represents the home range average for males and females combined, as Reid et al. [8] did not report estimates for the different sexes.

Mean annual 95% KUD home ranges for females were 21.0 ± SE 1.7 km^2^; for males it was 19.0 ± SE 1.3 km^2^. Core 50% KUD areas for females were 5.0 ± SE 0.5 km^2^; for males it was 4.7 ± SE 0.5 km^2^. Mean annual 90% KUD estimates were 20% smaller (females: 16.8 ± SE 1.4 km^2^; males: 15.1 ± SE1.1 km^2^) than the 95% KUD on average (Fig 6). Mean annual 95% MCPs were almost 50% smaller than 95% KUDs (see Fig 1).

Mean 95% MCP home ranges over the dry season for the females were 9.1 ± SE 1.0 km^2^ (n = 19) and 4.2 ± SE 0.6 km^2^ for the males (n = 6), and wet season for females were 6.2 ± SE 1.8 km^2^ (n = 6) and 8.2 ± SE 2.4 km^2^ for males (n = 6).

The ratio of 50 to 95% KUDs was 0.24 ± 0.01, indicating that black rhino spent half their time in just 24% of their annual home range. The ratio of use was similar between the sexes (male 0.24 ± SE 0.02, female 0.25 ± SE 0.01).

## Discussion

The historical sequence of home range size estimates for black rhino in HiP have been used to conclude that their habitat is deteriorating and the reserve over-populated because home ranges sizes have increased [8, 47]. But comparing KUD home ranges, constructed with a small number of locations collected fortuitously over many years, with more rudimentary home range procedures (e.g., 1-km^2^ grid-cell occupancy and MCPs) from data collected over shorter periods, is another explanation for the apparent increase [3]. We are now able to furnish our concerns with evidence for how the methods used have impacted home range size estimates and trends when comparing black rhino home range estimates in HiP.

### Building home ranges

Our estimates for the number of locations required to build seasonal and annual MCP home ranges are similar to the results of another study (i.e., ≥ 40 per annum, [6]). Home range studies of black rhino should not use fewer than 30 and 25 locations per wet and dry season, or 30 to 55 locations for annual home ranges, depending on rhino sex and the home range estimation technique used. Estimating male and MCP home ranges required greater numbers of locations than female and 90% KUD ranges, respectively. Black rhino are polygynous breeders [52], where a dominant bull actively displaces other males from their core range, while overlapping the ranges of multiple females (3 or more) that group together in multi-year associations [6]. Thus, males are probably more mobile than females, especially during the wet season and require greater numbers of locations to reliably describe their annual ranging. In previous studies as few as six [7, 53] and 10 [8] rhino locations have been used to estimate annual home range size in HiP. It is unlikely that those ranges are accurate or can be reliably compared with other estimates.

Estimating the seasonal home ranges required fewer locations than estimating the annual home ranges and wet season home ranges required the least number of locations. Estimating dry season ranges may require more locations because water access is limited to fewer sites and forage quality is poorer, thus motivating rhinos to make greater movements between sequential locations. Generally, more than 25 locations per 6-month season were required, but more than 30 per season would be unnecessary.

### Annual and seasonal home range sizes

Seasonal home ranges are seldom reported for black rhino [25], probably because the numbers of locations determined across each season are seldom sufficient. Interestingly, the largest average home range for each sex occurred in different seasons—males in the wet season and females in the dry season—and probably reflects differing relationships between resource availability (i.e., water and forage) and breeding activity. Females may move less during the wet season due to the greater availability of water and forage. Males, however, are likely to move more during the wet season when most conceptions occur [14].

The largest seasonal home ranges were similar to the size of annual home ranges (average male wet season MCP range size was 94% of their annual range, and female dry season ranges 88%), reflecting the importance of seasons as drivers of annual black rhino home ranges. Dry season home ranges are likely to be more useful for understanding the resource limits of reserve habitat for female black rhino and wet season home ranges more useful for understanding the spatial requirements of breeding males. The design and management of protected areas for black rhino could benefit from applying this understanding about the different seasonal use of space by the sexes.

Our ratios of total annual range to core range use (24% of the home range used 50% of the time) were similar to those reported by others (references [6] and [54]: 21% and 23% of the home range, respectively). Compared to other animals (e.g., horses 12%, [34]; and spotted turtles 8%, [55]), rhino appear to use the central portions of their home ranges less intensively—perhaps a reflection of their larger body size and dependence on larger amounts of lower quality forage [56].

The 95% MCPs and 90 and 95% KUDs using the same location data produced very different estimates of annual home range size. Average annual 95% KUD home range estimates were more than twice as large as 95% MCP home range sizes, primarily due to the interpretation of density around peripheral locations and the smoothing parameter used (Fig 1). Our 90% KUD home ranges were 74% larger than the MCPs. Others have found similar discrepancies between home range procedures [4]. Black rhino home ranges derived using KUDs cannot be legitimately compared with more rudimentary techniques like MCPs or 1-km^2^ grid-cell occupancy in the way they have been (e.g., [8], [47]; see Table 1 and Fig 6).

### Kernel UDs and MCPs compared

Our 95% KUD home range estimates using larger numbers of animal locations (average 51 ± SE 3.3 locations) from a single year are 12% smaller (2.7 km^2^) than the average of those presented previously ([8]: i.e., 20.4 km^2^ cf. 23.0 km^2^; see Fig 6) that include as few as 10 locations collected fortuitously across 11 years (Table 1). The adaptive KUD methods used by Reid et al. [8] are also known to consistently overestimate the area of the distribution compared to the fixed bivariate KUD techniques recommended [57] and adopted in this study. When 90% KUDs are used to avoid the errors inherent in 95% KUDs [40], our average annual ranges are 16.2 km^2^ and so 30% smaller than Reid et al. [8] estimates. This comparison indicates the degree to which Reid et al. [8] home range sizes might be inflated by multi-year data collection and small sample size that generate a greater proportionate number of peripheral locations with large adaptive KUDs around them.

Although, as predicted, our range size estimates were smaller than those reported previously [8, 47], we expected their use of small amounts of location data spread across multiple years [3, 25], to result in a much larger home range inflation. It may be that we have underestimated the degree of home range inflation because our bivariate KUD home range estimates are also somewhat inflated by spatial error from our triangulations which contribute to about 50% of our animal locations. However, a retrospective comparison of rhino home ranges using our triangulation and direct sightings datasets yielded only a small (2.8%) difference in their range sizes compared to their combined use. Annual 95% KUD ranges using only triangulation and then only sightings data yielded average ranges that were 18.0 versus 17.5 km^2^, respectively. It is more likely, therefore, that the numbers of locations Reid et al. [8] used are so small (as few as 10) that they under-estimated multi-year range sizes but, nevertheless, approximated rhinos’ smaller annual home range sizes. Indeed, when Slotow et al. [47] re-analysed the same 11-year data set as Reid et al. [8] using rhino (n = 19) that had a sufficient number of locations (i.e., ≥ 50), average range sizes became 68% larger than our estimate (i.e., 20.4 km^2^ cf. 34.3 km^2^). Thus, even with enough locations, collecting them fortuitously over protracted time periods (*c*. 5 locations per annum over 11 years) will significantly inflate home range size estimates for black rhino. Slotow et al.’s [47] re-analysis also suggests that established populations, like the one in HiP, undergo some degree of inter-annual home range variation over a decade, as has been reported in an expanding black rhino population (e.g., Great Fish River Reserve, South Africa, [6]).

Average annual MCP home range estimate for all rhino was 9.8 km^2^, which is 19.5% (2.2 km^2^) larger than Hitchin’s [46] estimate (7.5 km^2^) from a radio-telemetry study in the same park 50 years earlier (Table 1 and Fig 6). Our home range size estimates are probably larger because Hitchins did not use MCPs in their strictest sense. Instead he drew a line around all peripheral location points in sequence i.e., approximating a type of maximum (cf. minimum) convex polygon, which produces ranges smaller than conventional MCPs from the same location points. When we applied Hitchin’s [45–46] technique to our location data we, nonetheless, generated similarly sized home ranges to those he estimated during the late-1960s (Fig 6).

The debate about whether small numbers of fortuitous locations of black rhino collected across multiple years can be used to construct reliable estimates of home range size for evaluating the status of the black rhino population or its habitat [3, 8, 25, 47] is resolved by our comparisons. Animal location data like that described in Reid and Slotow et al. [8, 47] should not be used to construct KUD home ranges because it will inflate home range size. Then, such KUD home ranges should not be compared with other more rudimentary techniques (e.g., 1-km^2^ grid-cell occupancy, [7, 53]; MCPs, [30, 35]). Importantly, these comparisons confirm that the home ranges for black rhino in HiP have not increased substantially over the last half century. Rather, MCP home ranges have probably always been about 9 km^2^ and KUD home ranges about 20 km^2^ in size.

### Population management implications

Previously, authors have claimed a substantial (54%) increase in black rhino home range size in HiP and attributed it to deteriorating habitat and over-population [8, 47]. These conclusions lent support to a policy for increased live harvest of black rhino from HiP [19–20], when others thought the population might have been over-harvested and in decline [16, 58]. Unfortunately, the conclusions were reached by comparing KUD estimates using as few as 10 locations collected across 11 years with values derived from a smaller, earlier portion of the same dataset and using more rudimentary techniques (Table 1). The comparison is flawed because, as we demonstrate, KUD techniques using the same data produce much larger home range estimates than previous techniques (e.g., KUD estimates were 53% larger than MCPs; Figs 1 and 3). Our evidence indicates that home range sizes amongst black rhino in HiP have not increased.

Home range sizes are routinely reported in Rhino Management Group (RMG: International African conservation agencies tasked with rhino meta-population monitoring and management) reports (e.g., [53, 59]). Average home range area estimates for many populations across Africa are regularly consulted by meta-population managers to determine reserve stocking (carrying capacity) and harvesting levels for black rhino populations [19, 59–62] by making the assumption that there is an inverse relationship between range size and resource density [7–8, 18–19, 47, 60–61]. Inaccurate, non-standardised, home range sizes, therefore, have the potential to mislead black rhino population management [3, 25].

Black rhino population home range sizes vary considerably across the African continent, with the smallest ranges reported in relatively wet and sub-tropical HiP (3 km^2^, [45]) and the largest in arid Namibia (e.g., 3, 000 km^2^, [63]). An understanding of what drives this variation eludes us because home range studies of black rhino have largely been data deficient and procedurally inconsistent (Table 1, S1 Table). Building a model for black rhino socio-spatial ecology and testing for the predicted inverse relationship between home range size and habitat quality that is responsive to changes in resource density [3, 15, 25] requires the standardisation of home range data and procedures. Black rhino home ranges should be defined over meaningful environmental cycles (i.e., a year or season), be calculated using 25 to 30 locations per season, or 30 to 55 per annum depending on rhino sex and technique applied, and comparisons of home ranges only made when similar techniques are used.

## Supporting Information

S1 Table Detailed comparison of methodology of black rhinoceros home range studies outside of Hluhluwe-iMfolozi Park. Note that this table excludes home range studies in unpublished material e.g., Rhino Management Group (RMG) reports. The RMG reports are not publicly available, but several reports seen by the authors list average home range sizes for most black rhino populations across Africa without reference to the methodology or the home range studies used to calculate them. * The average duration (time-period) between locations per rhino.
(PDF)
