# Supplementary material for: Standardising Home Range Studies for Improved Management of the Critically Endangered Black Rhinoceros
Source: PLoS One. 2016 Mar 30;11(3):e0150571. doi: 10.1371/journal.pone.0150571 (PMC4814105; doi:10.1371/journal.pone.0150571)
Supplement: S1 Table — Note that this table excludes home range studies in unpublished material e.g., Rhino Management Group (RMG) reports. The RMG reports are not publicly available, but several reports seen by the authors list average home range sizes for most black rhino populations across Africa without reference to the methodology or the home range studies used to calculate them. * The average duration (time-period) between locations per rhino. (PDF) [file pone.0150571.s001.pdf]

**Data Collection**	**Data Analysis**	**Obser. Period**	**Locations per rhino**	**Sampling frequency***	**Focal population**	**Reported home range size (km^2)**	**Location**	**Studies**
Ground search (GS), fortuitous observations (FO)	Visual approximation using all points (VA)	*c.* 1963-1965	Not reported	Not reported	1m, 3f	14	Ngorongoro Crater, Tanzania	Klingel and Klingel 1966 [1]
GS, FO	VA	*c.* 1964-1966	2-16	Not reported	4m, 4f	11.7	Ngorongoro Crater, Tanzania	Goddard 1967 [2]
GS (incl. car), FO	VA	c. 1967-1968	Not reported	Not reported	12m, 14f	7.8	Nairobi NP, Kenya	Hamilton and King 1969 [3]
GS, FO	VA	Jun 1966-c. 1968	3-44	Not reported	9m, 8f	31-41	Otjovasandu, Namibia	Joubert and Eloff 1971 [4]
GS, FO	Grid occupancy data (GOD) using all points (1km^2 grid size)	3-months (May 1971-Aug. 1972)	Not reported	Not reported	5m, 7f	5.6-22.7	Masai Mara, Kenya	Mukinya 1973 [5]
FO	VA	Feb 1974-Jan 1978	Not reported	Not reported	67	88	Serengeti National Park & Ngorongoro Conservation Area, Tanzania	Frame 1980 [6]
GS, FO	VA	*c.* 1978- 84	Not reported	Not reported	Not reported	3, 000	Damaraland & Skeleton Coast Park, Namibia	Loutit 1984 [7]
Not reported	Not reported	Not reported	Not reported	Not reported	6m	28.8	Laikipia Plateau, Kenya	Brett et al. 1989 [8]
GS, FO	GOD (1km^2 grid size)	Jul 1984-86	$\geq$ 10	Not reported	2m, 2f	11.4	Ndumo GR, South Africa	Conway & Goodman 1989 [9]
FO (concurrent with other studies on population)	VA plotted on 1:50,000 maps with 1cm^2 grid (=0.25 km^2 area)	Annual and seasonal (wet: Jan-Jun. & dry: Jul-Dec 1980-May 1982	Not reported	Not reported	2m, 7f	Annual: 45.9 (m), 27.7 (f); wet: 23 (f), 36.8 (m), dry: 36.8 (m), 20.6 (f)	Ngorongoro Crater, Tanzania	Kiwia 1989 [10]
FO	100% MCP	1992- 1995	Not reported	Not reported	7m, 13f	42 (m), 51 (f)	Masai Mara, Kenya	Morgan-Davies 1996 [11]

Data Collection	Data Analysis	Obser. Period	Locations per rhino	Sampling frequency*	Focal population	Reported home range size (km^2)	Location	Studies
GS, FO	VA to nearest 500m (276 data points); MCP	Not reported	Males= 12.0 +1.87 [SE] ; Females= 12.9 + 1.71).	Not reported	9m, 13f	730 ± 209 SE (m); 538 ± 161 SE	Kaokoveld, Kunene Province, Namibia	Berger 1997 [12]
GS, annual helicopter census	VA, excluded steep hill areas	c. 1982-1989	Not reported	Not reported	Not reported	28 (m), 52 (f)	Pilanesberg NP, North-West Prov., South Africa	Adcock et al. 1998 [13]
FO	100% MCP	1982-2002	Not reported	Not reported	7f	94.4-441.2	Kunene and Erongo, region, Namibia	Hearn et al. 2000 [14]
GS	95% MCP; harmonic isopleths, 70% cluster polygons	3-months (Jul-Sep 1995)	Not reported	Not reported	6m, 6f	2.3-14.4	Sweetwaters Rhino Sanctuary, Laikapia District, Kenya	Tatman et al. 2000 [15]
GS	Figures showing plotted locations	Annual (Nov 1999 – Nov 2000)	Not reported	Not reported	1m	Not reported	Southern Timbavati, Lowveld, South Africa	Roche 2001 [16]
FO	100% MCP	1982-2002	Not reported	Not reported	7f	94.4-441.2	Kunene and Erongo, region, Namibia	Hearn et al. 2000 [14]
GS	95% MCP; harmonic isopleths, 70% cluster polygons	3-months (Jul-Sep 1995)	Not reported	Not reported	6m, 6f	2.3-14.4	Sweetwaters Rhino Sanctuary, Laikapia District, Kenya	Tatman et al. 2000 [15]
GS	Figures showing plotted locations	Annual (Nov 1999 – Nov 2000)	Not reported	Not reported	1m	Not reported	Southern Timbavati, Lowveld, South Africa	Roche 2001 [16]
FO	100% MCP	1982-2002	Not reported	Not reported	7f	94.4-441.2	Kunene and Erongo, region, Namibia	Hearn et al. 2000 [14]
GS	95% MCP; harmonic isopleths, 70% cluster polygons	3-months (Jul-Sep 1995)	Not reported	Not reported	6m, 6f	2.3-14.4	Sweetwaters Rhino Sanctuary, Laikapia District, Kenya	Tatman et al. 2000 [15]
GS	Figures showing plotted locations	Annual (Nov 1999 – Nov 2000)	Not reported	Not reported	1m	Not reported	Southern Timbavati, Lowveld, South Africa	Roche 2001 [16]

Data Collection	Data Analysis	Obser. Period	Locations per rhino	Sampling frequency*	Focal population	Reported home range size (km^2)	Location	Studies
GS, FO	GOD (6ha grids, 95% MCP (annual); 50% core adaptive kernel (seasonal)	Annual and seasonal from 1992-1997	≥ 35	Not reported	6m, 6f	11.7 (annual: 95% MCP) 6.8 (seasonal: 50% kernel)	Great Fish River Reserve, Eastern Cape, South Africa	Lent and Fike 2003 [17]
Radio-telemetry (R-T), FO	70% Harmonic mean to calculate core areas	Seasonal between Mar 1998-Jul 2000	4-8	Not reported	2m, 3f (3 hand reared, 2 wild)	4.1-8	Matsudona National Park, Zimbabwe	Matipano 2004 [18]
R-T	100% MCP, 50 and 95% core adaptive kernel	Ranging from 494-674 days (Mar 2005-Feb 2007)	147-306	≥ weekly	3m, 3f	100%MCP: 86.8(m), 140.1(f)	Rhino Custodianship Scheme Reserve, Adjacent to Etosha NP, Namibia	Göttert et al. 2010 [19]
R-T (not random stratified approach)	Annual: 100% MCP and 50% and 95% Local Convex Hull	Annual (Jan 2006 –Dec 2006)	38-54	4 sightings per rhino per month	8f, 9m	100% MCP: 12.3; 50% kernel: 0.9; 95% kernel: 3.77	Zululand Rhino Reserve, KwaZulu-Natal, South Africa	Odendaal – Holmes et al. 2014 [20]

References

1. Klingel H, Klingel U. The rhinoceros of Ngorongoro Crater. Oryx. 1966; 8: 302-306.
2. Goddard J. Home range, behaviour, and recruitment rates of two black rhinoceros populations. Afr J Ecol. 1967; 5: 133-150.
3. Hamilton PH, King JM. The fate of black rhinoceroses released in Nairobi National Park. Afr J Ecol. 1969; 7: 73-83.
4. Joubert E, Eloff FC. Notes on the ecology and behaviour of the black rhinoceros *Diceros bicornis* Linn. 1758 in South West Africa. Madoqua. 1971; 1: 5-53.
5. Mukinya JG. Density, distribution, population structure and social organization of the black rhinoceros in Masai Mara Game Reserve. Afr J Ecol. 1973; 11: 385-400.
6. Frame GW. Black rhinoceros (*Diceros bicornis* L.) sub-population on the Serengeti Plains, Tanzania. Afr J Ecol. 1980; 18: 155-166.
7. Loutit BD. A study of the survival means of the black rhino in the arid areas Damaraland and Skeleton Coast Park. Quagga. 1984; 7: 4-5.
8. Brett RA, Hodges JK, Wanjohi E. Assessment of reproductive status of the black rhinoceros (*Diceros bicornis*) in the wild. Symp Zool Soc Lond. 1989; 61:147-161.

9. Conway AJ, Goodman PS. Population characteristics and management of black rhinoceros *Diceros bicornis minor* and white rhinoceros *Ceratotherium simum simum* in Ndumu Game Reserve, South Africa. Biol Cons. 1989; 47: 109-122.
10. Kiwia HYD. Ranging patterns of the black rhinoceros (*Diceros bicornis* L.) in Ngorongoro Crater, Tanzania. Afr J Ecol. 1989; 27: 305-312.
11. Morgan-Davies M. Status of the black rhinoceros in Masai Mara National Reserve, Kenya. Pachyderm. 1996; 21: 38-45.
12. Berger J. Population constraints associated with the use of black rhinos as an umbrella species for desert herbivores. Cons Biol. 1997; 11: 69-78.
13. Adcock KH, Hansen B, Lindemann H. Lessons from the introduced black rhino population in Pilanesberg National Park. Pachyderm. 1998; 26: 40-51.
14. Hearn ME, Loutit BD, Uri-Khob S. The black rhinoceros of north-western Namibia (*Diceros bicornis bicornis*): the role of density dependence and its management implications. Namibia Sci Soc J. 2000; 48: 11-39.
15. Tatman SC, Stevens-Wood B, Smith VBT. Ranging behaviour and habitat usage in black rhinoceros, *Diceros bicornis*, in a Kenyan sanctuary. Afr J Ecol 2000; 38: 163-172.
16. Roche C. Preliminary findings of home range, diet and midden significance in a black rhino bull. CCA Ecological Journal 2001. 3: 29-33.
17. Lent PC, Fike B. Home ranges, movements and spatial relationships in an expanding population of black rhinoceros in the Great Fish River Reserve, South Africa. S Afr J Wild Research. 2003; 33: 109-118.
18. Matipano G. Black rhinoceros mortality in Matusadona National Park, Zimbabwe: 1992–2003. IUCN report. 2004: pp.109.
19. Göttert T, Schone J, Zinner D, Hodges JK, Boer M. Habitat use and spatial organisation of relocated black rhinos in Namibia. Mammalia. 2010; 74: 35-42.
20. Odendaal-Holmes K, Marshal JP, Parrini F. Disturbance and habitat factors in a small reserve: space use by establishing black rhinoceros (*Diceros bicornis*). S Afr J Wild Res. 2014. 44: 148-160.
